# Supplementary material for: Hypoxia-induced mitochondrial abnormalities in cells of the placenta
Source: PLoS One. 2021 Jan 12;16(1):e0245155. doi: 10.1371/journal.pone.0245155 (PMC7802931; doi:10.1371/journal.pone.0245155)
Supplement: S1 Fig — No significant differences were found for LDH activity between the control- and hypoxic-conditioned villous explant medium indicating that viability of the placental villous explants was comparable between the two conditions after 3 h of incubation. Furthermore, when BeWo cells were cultured for 6 h, 24 h and 48 h under a normoxic or hypoxic condition, only at 48 h a significant increase in LDH activity was observed in cell supernatant of BeWo cells exposed to hypoxia compared to the control condition (S2 Fig). Since cell viability in the hypoxic condition significantly decreased after 48 h culturing under hypoxia, compared to the control condition, and therefore affecting the readouts of our study, 24 h exposure to hypoxia was used in this study. (PDF) [file pone.0245155.s001.pdf]

Blot I BeWo cells (3 replicates, 1st experiment)

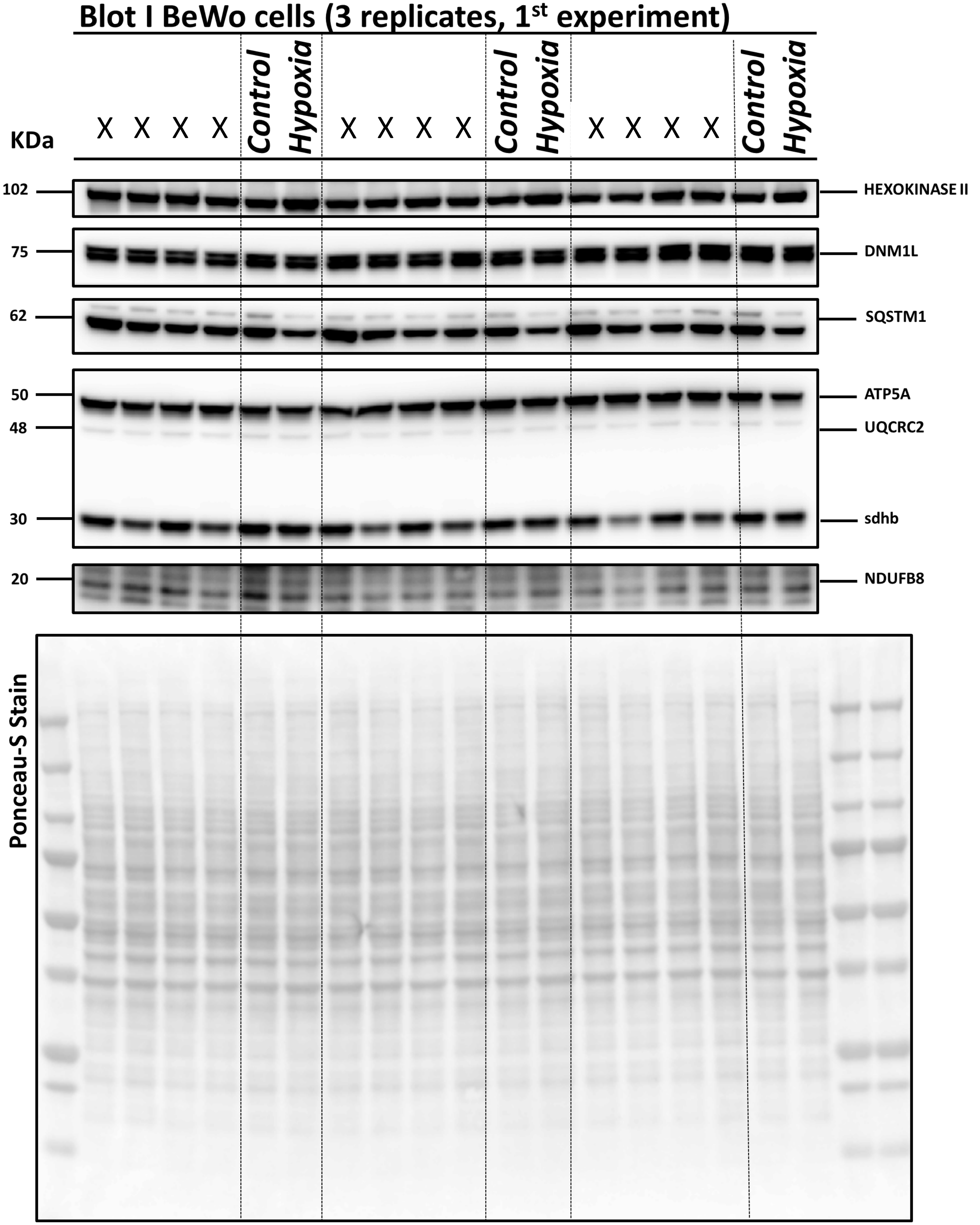

X: Conditions not relevant to for this research paper

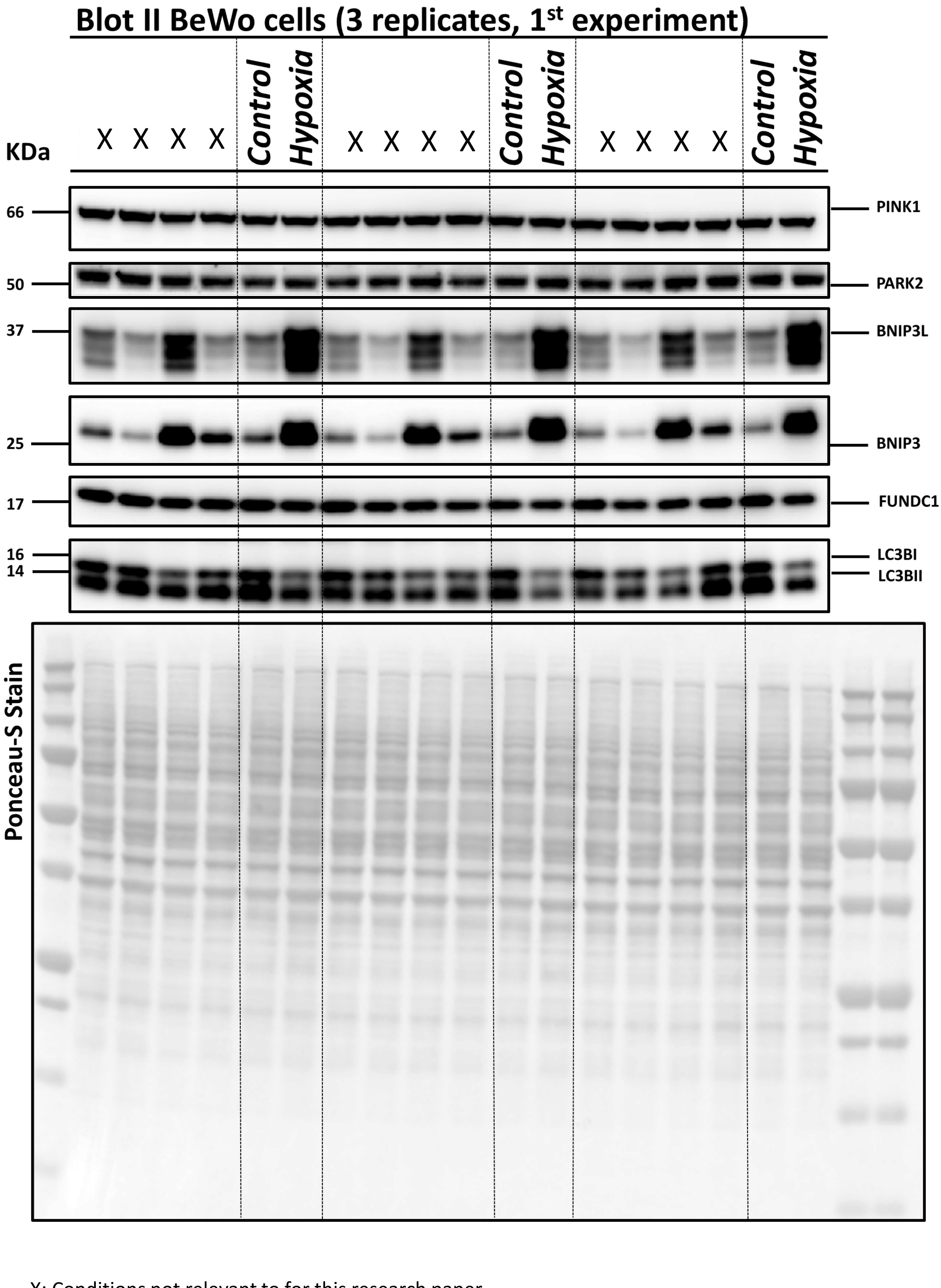

X: Conditions not relevant to for this research paper

Blot III BeWo cells (3 replicates, 1st experiment)

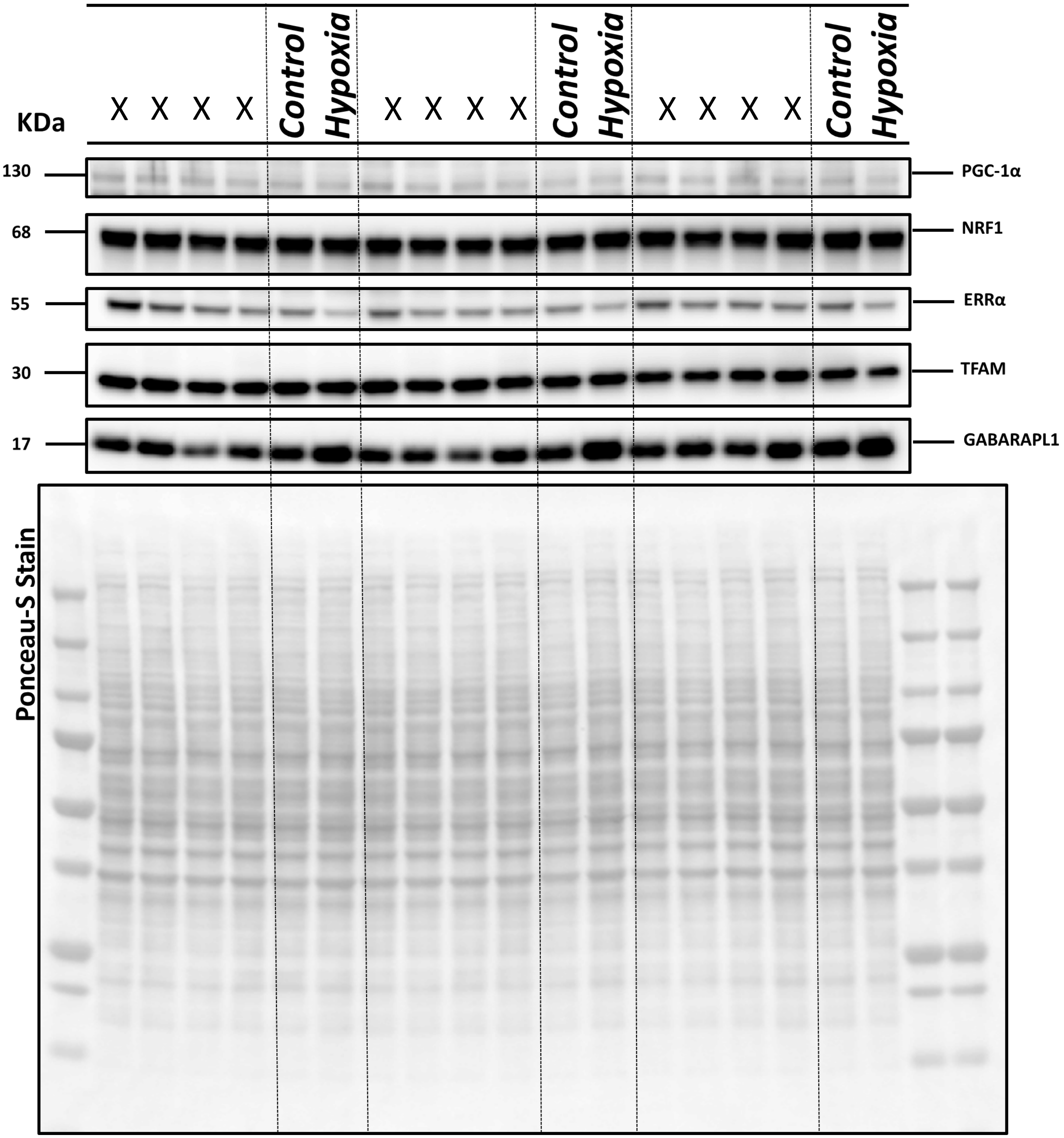

X: Conditions not relevant to for this research paper

Blot IV BeWo cells (3 replicates, 1st experiment)

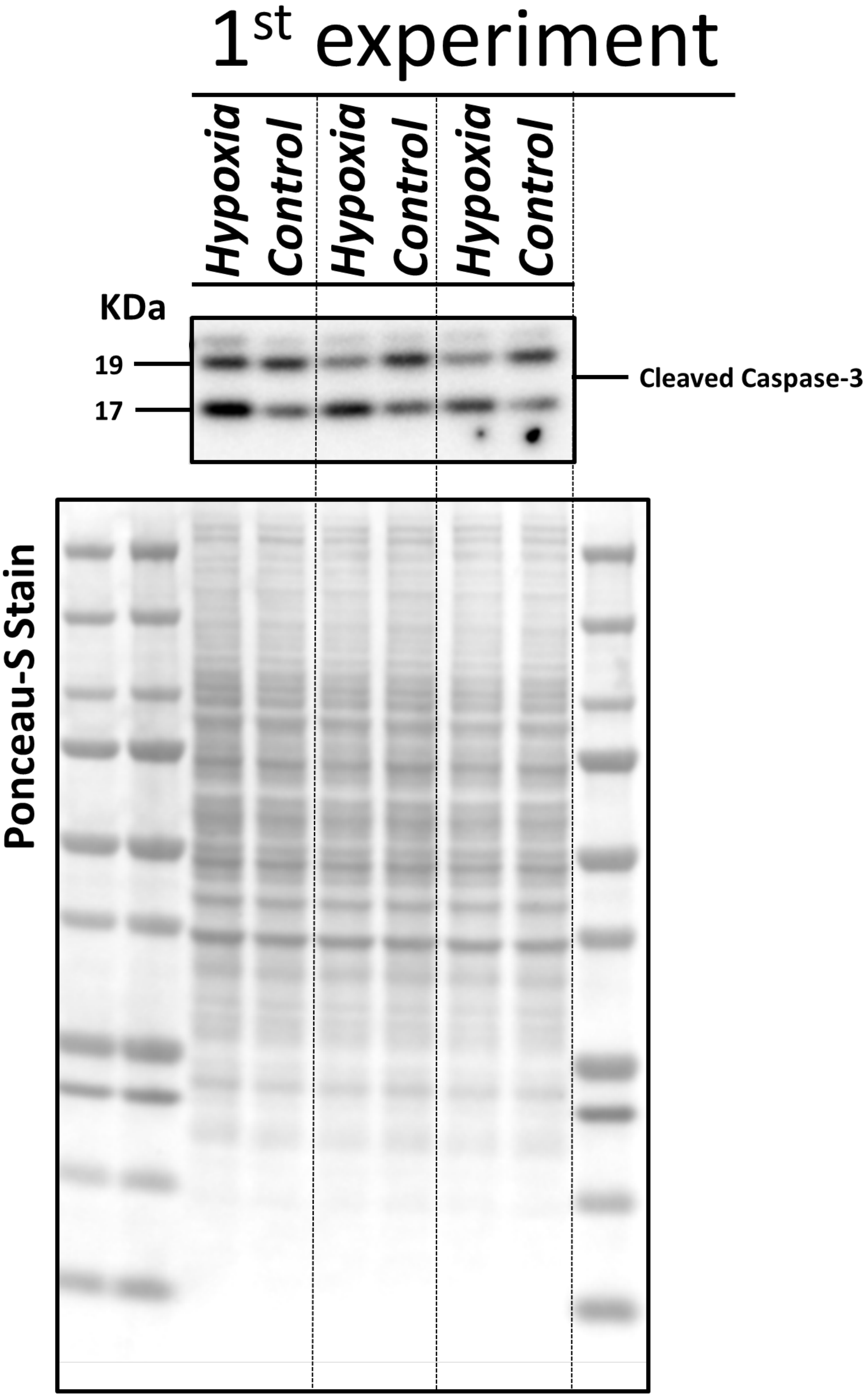

Blot I BeWo cells (6 replicates, 2nd and 3th experiment)

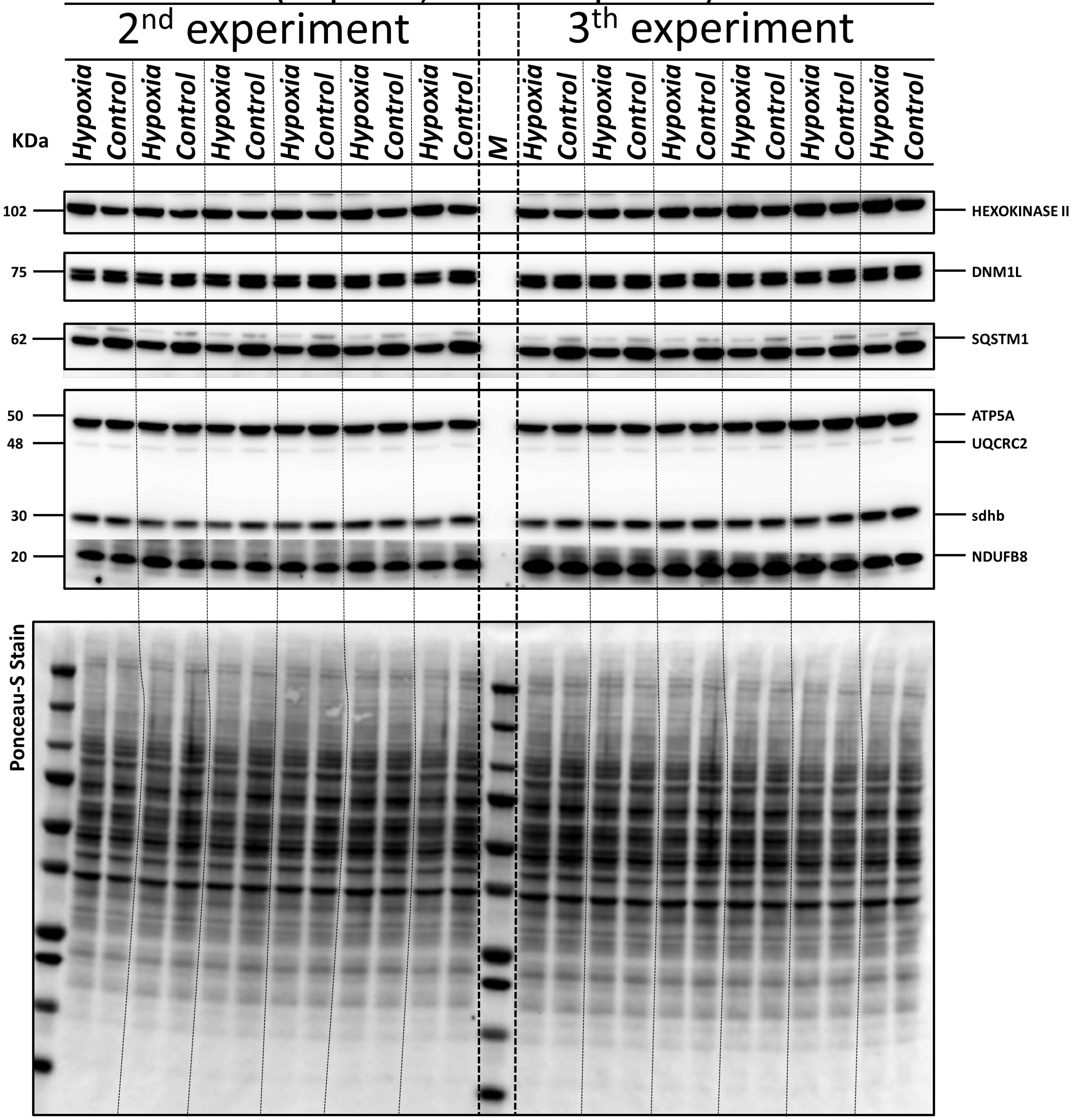

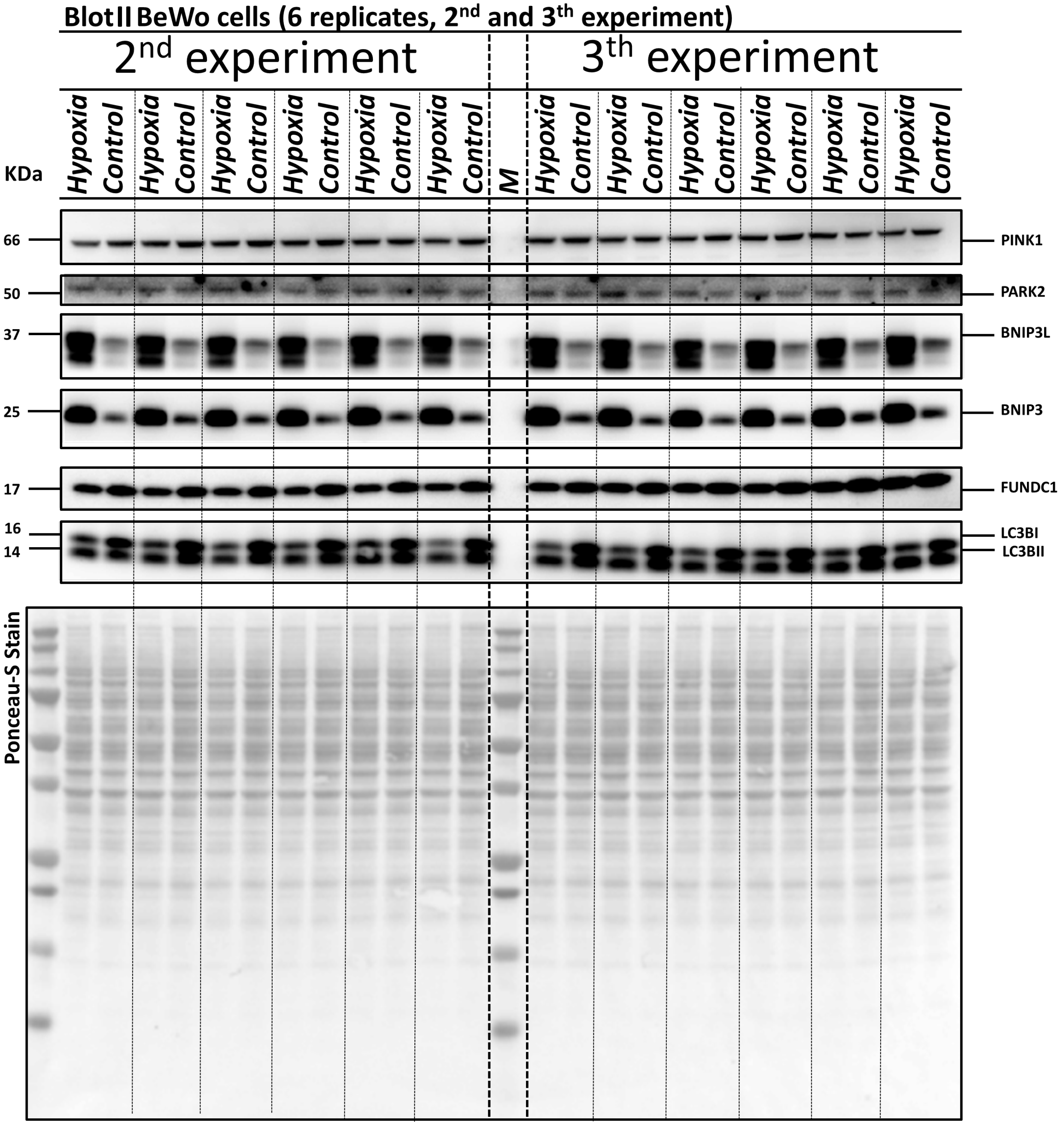
Blot II BeWo cells (6 replicates, 2nd and 3th experiment)
2nd experiment
3th experiment
KDa
Hypoxia Control Hypoxia Control Hypoxia Control Hypoxia Control Hypoxia Control Hypoxia Control
M
Hypoxia Control Hypoxia Control Hypoxia Control Hypoxia Control Hypoxia Control Hypoxia Control
66
PINK1
50
PARK2
37
BNIP3L
25
BNIP3
17
FUNDC1
16
14
LC3BI
LC3BII
Ponceau-S Stain

Blot III BeWo cells (6 replicates, 2nd and 3th experiment)

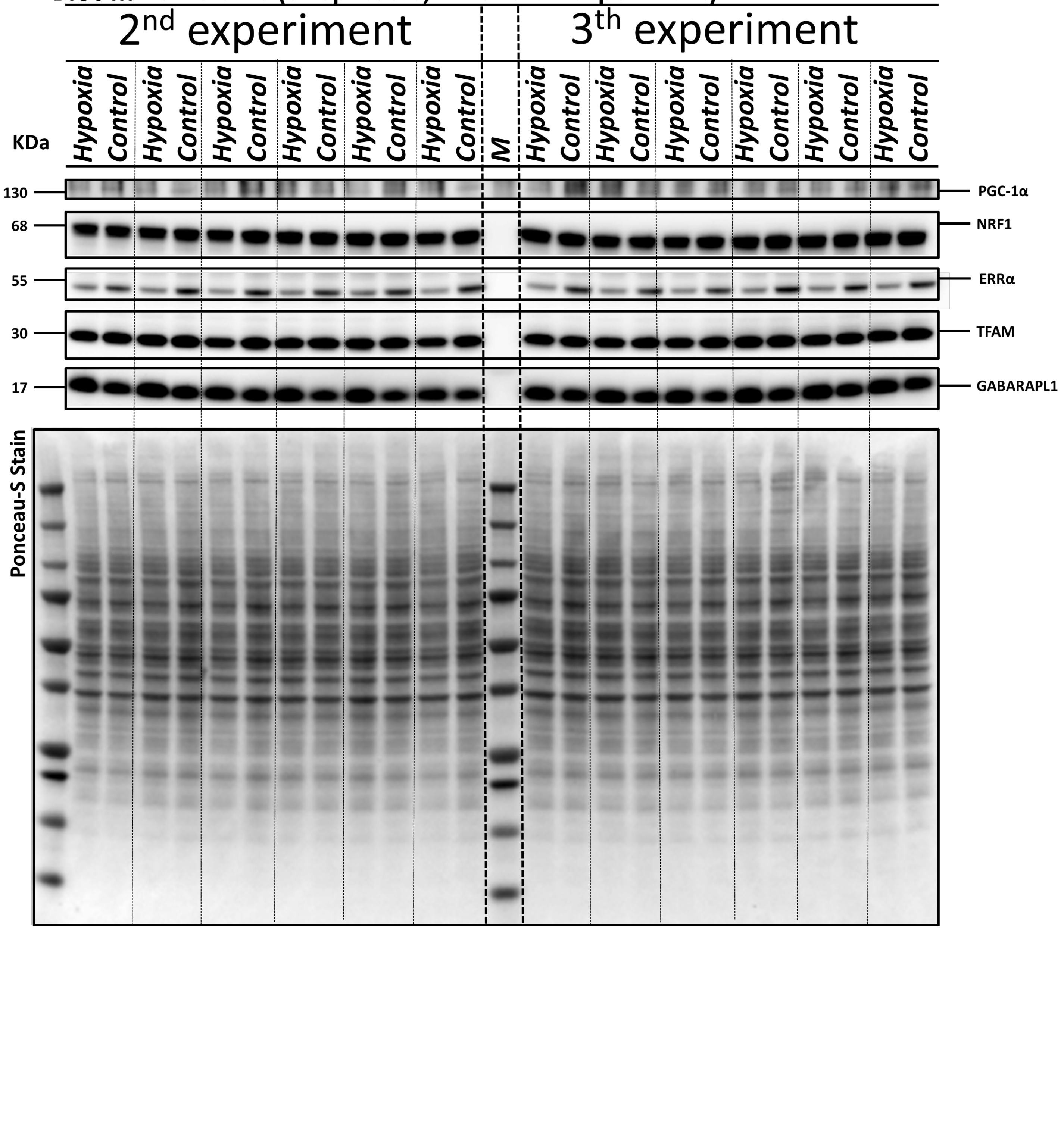

Blot IV BeWo cells (6 replicates, 2th and 3th experiment)

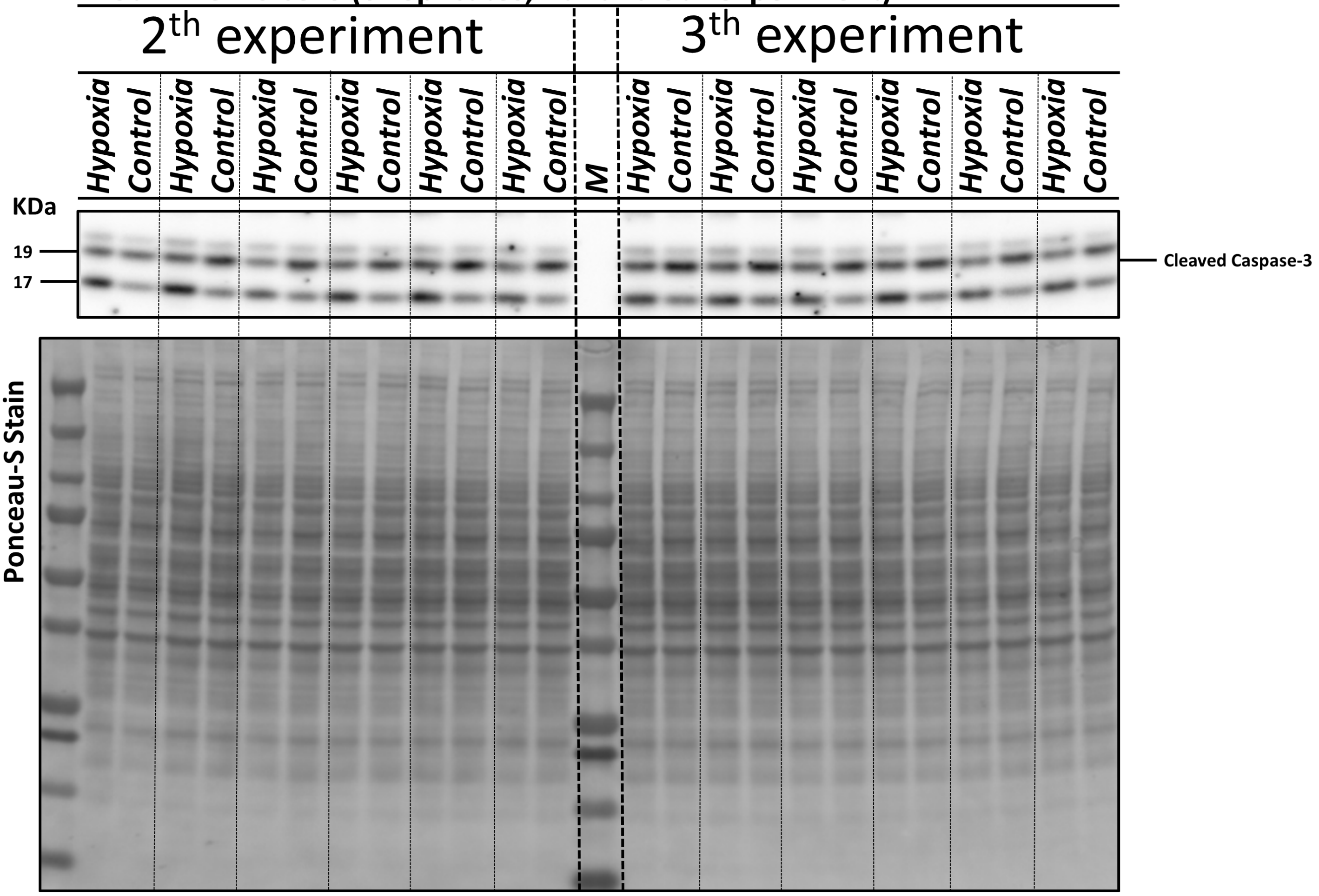

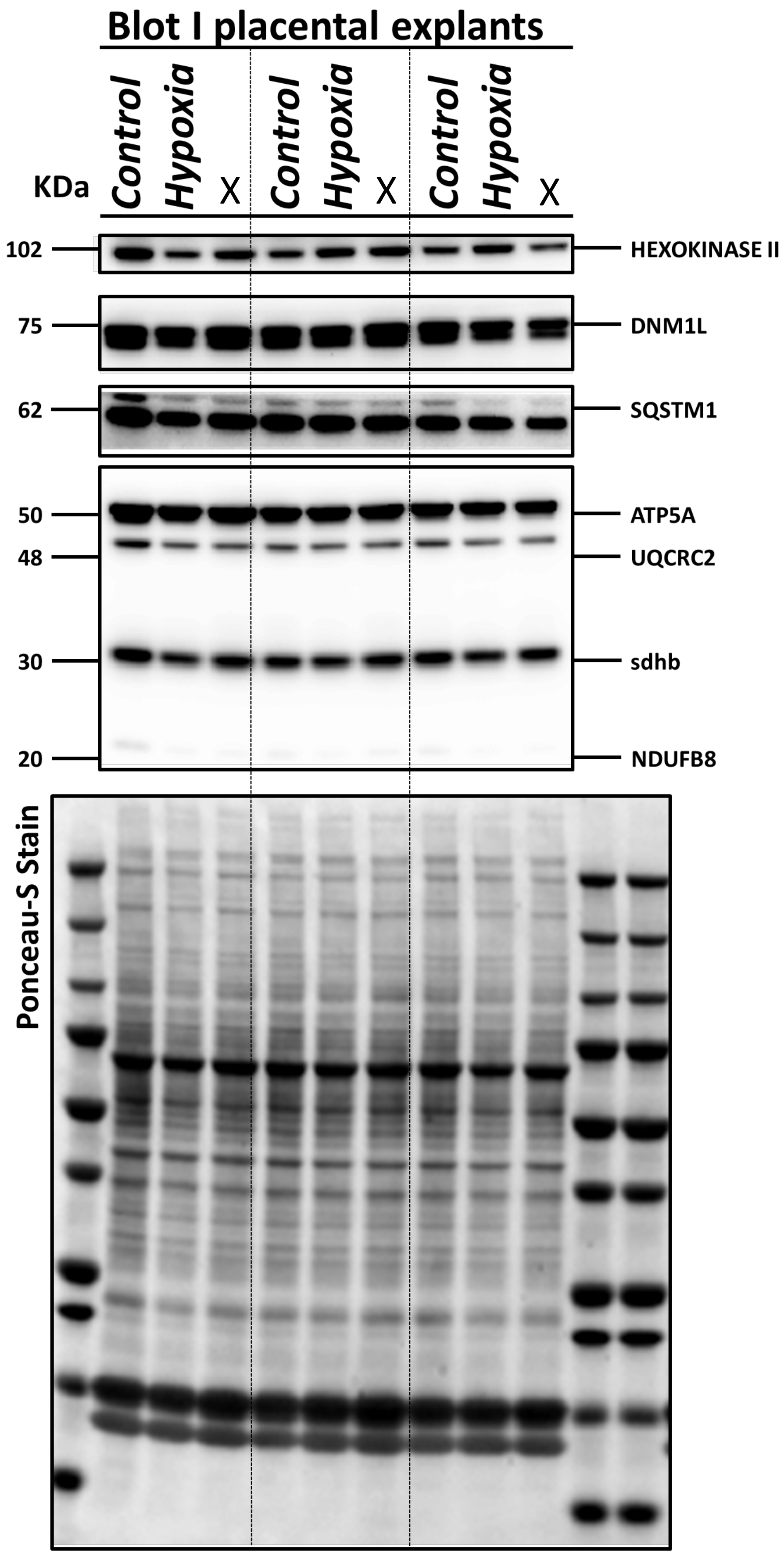

X: Conditions not relevant to for this research paper

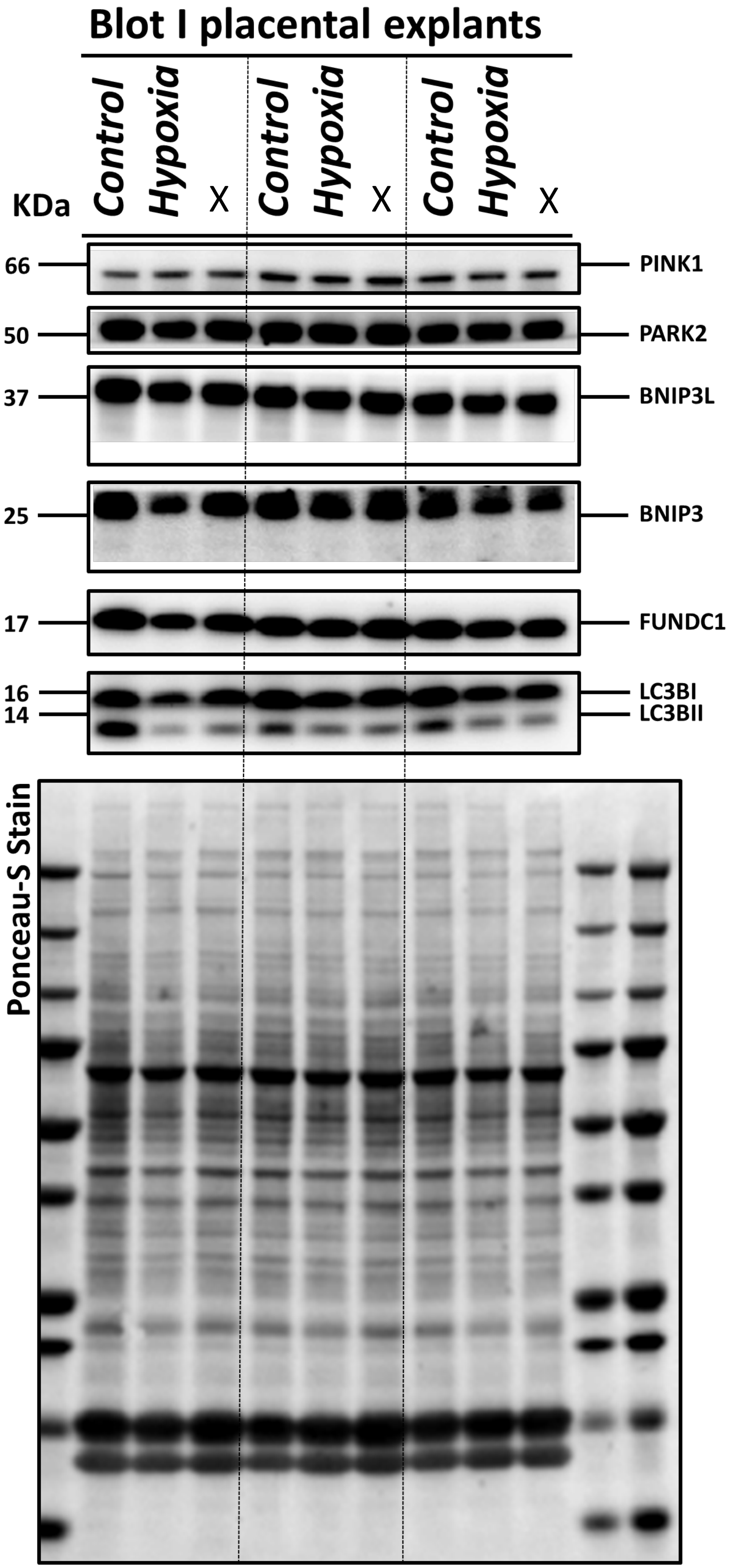

X: Conditions not relevant to for this research paper

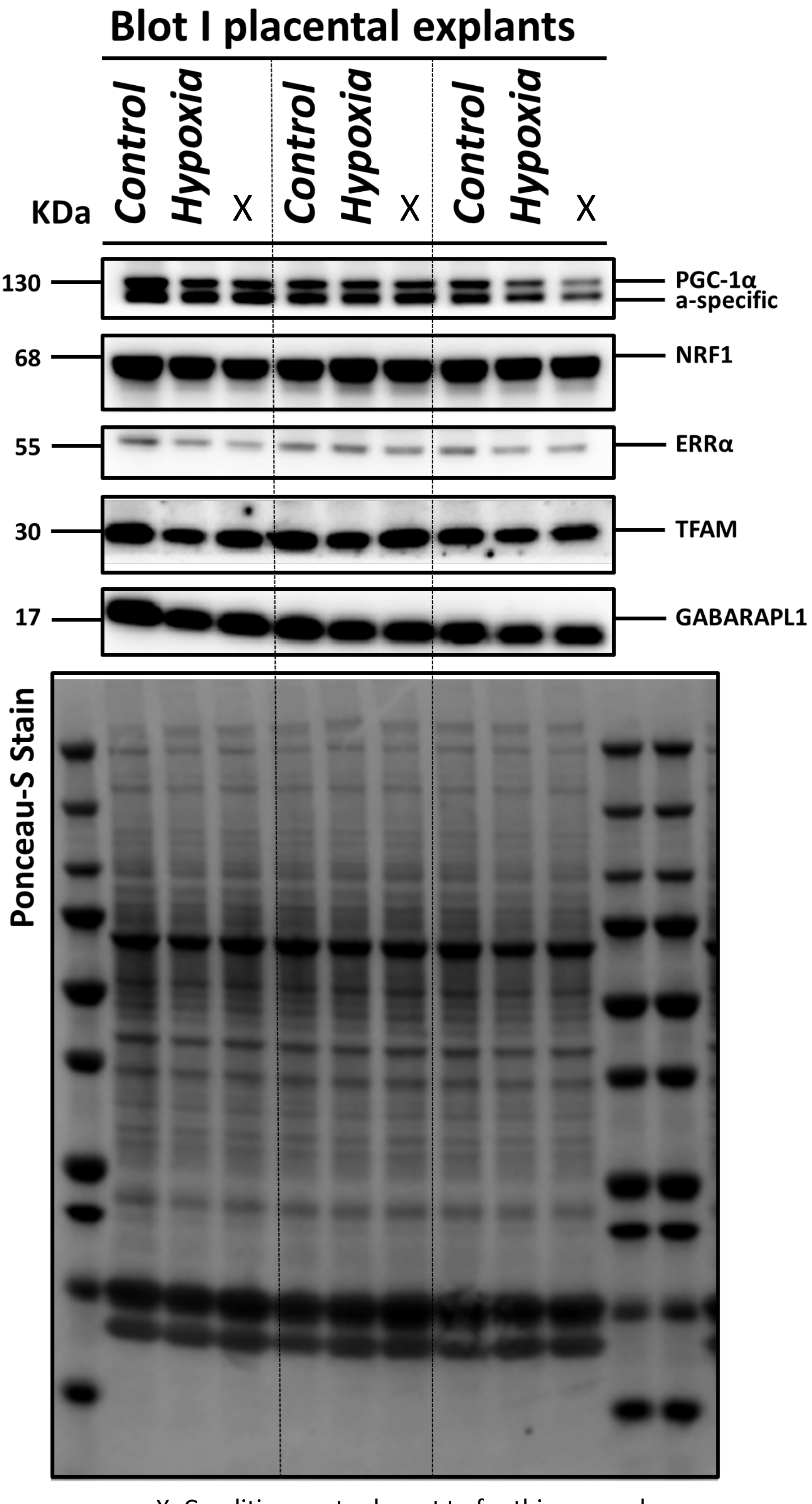

X: Conditions not relevant to for this research paper
