# Supplementary figures and images for: Hypoxia-induced mitochondrial abnormalities in cells of the placenta
Source: PLoS One. 2021 Jan 12;16(1):e0245155. doi: 10.1371/journal.pone.0245155 (PMC7802931; doi:10.1371/journal.pone.0245155)

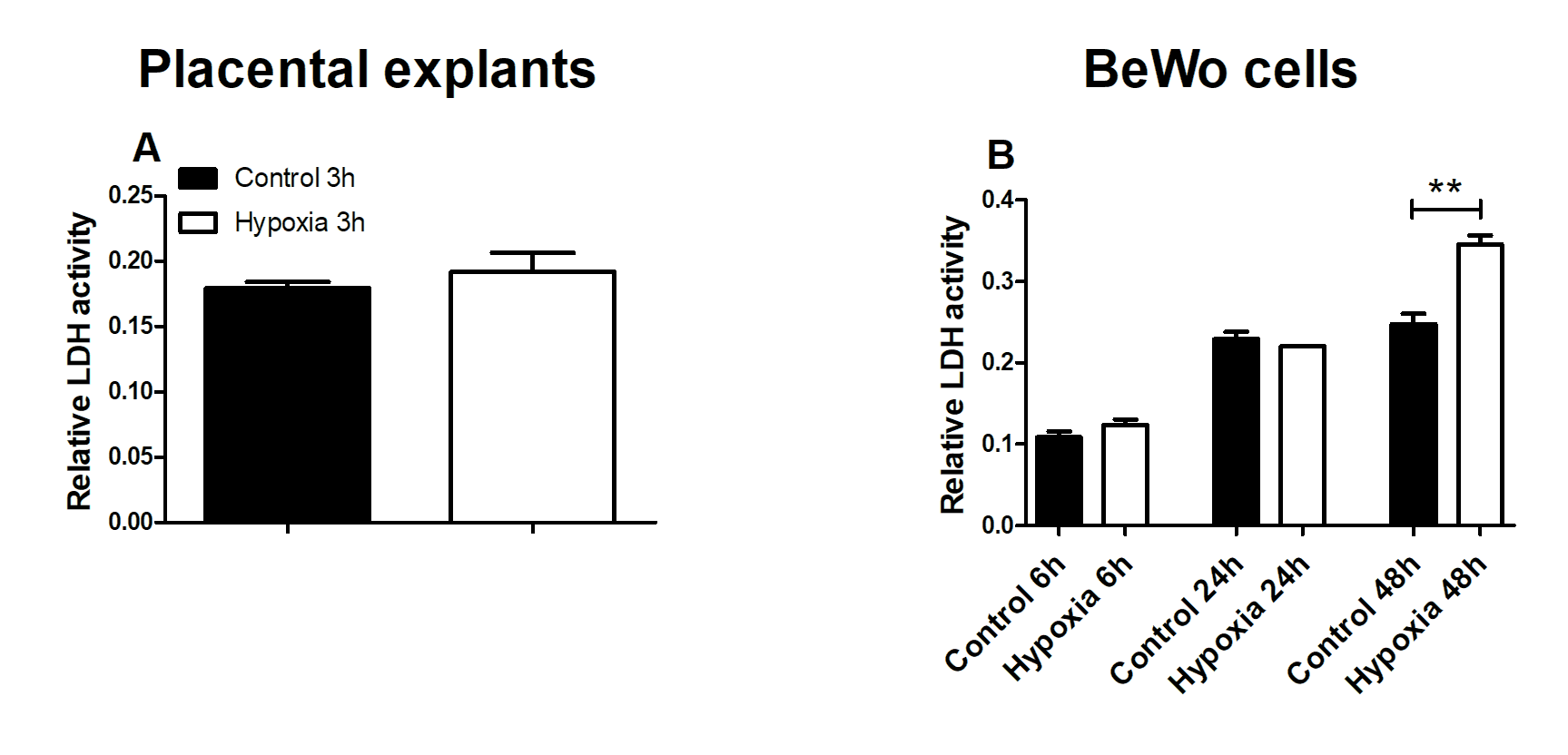

Supplement: S2 Fig — LDH activity between the control- and hypoxic-conditioned villous explant after 3 h of incubation (A) and in BeWo cells after 6 h, 24 h and 48 h incubation (B). To confirm whether mitochondria are a significant source for ROS in placentae upon hypoxia, the effect of the mitochondrially targeted antioxidant MitoQ and the systemically acting antioxidant quercetin on ROS formation and oxidative status was tested. Intracellular ROS levels increased more than two-fold in trophoblasts exposed for 24 h to hypoxia compared to normoxia-exposed trophoblasts (S3A Fig). Moreover, the mitochondrially targeted antioxidant MitoQ (4 and 8 μM) ameliorated hypoxia-induced intracellular ROS formation in trophoblasts in a concentration-dependent manner. The systemically acting antioxidant quercetin (3 μM), did not induced significant reductions in intracellular ROS formation during exposure to hypoxia. Interestingly, the combination of the lowest concentration of MitoQ (1 μM) and quercetin (3 μM) significantly decreased intracellular ROS production upon 24 h exposure to hypoxia and was the only condition that was not significantly different from the normoxic condition (S3A Fig). In agreement, the hypoxia-induced increase of the GSSG/GSH ratio, could be normalized by MitoQ (4 and 8 μM) and quercetin (3 μM), but were still significantly different compared to the control condition (S3B Fig). Interestingly, the combination of the lowest concentration of MitoQ (1 μM) and quercetin (3 μM), normalized the GSSG/GSH ratio during exposure to hypoxia (S3B Fig). (TIF) [file pone.0245155.s002.tif]

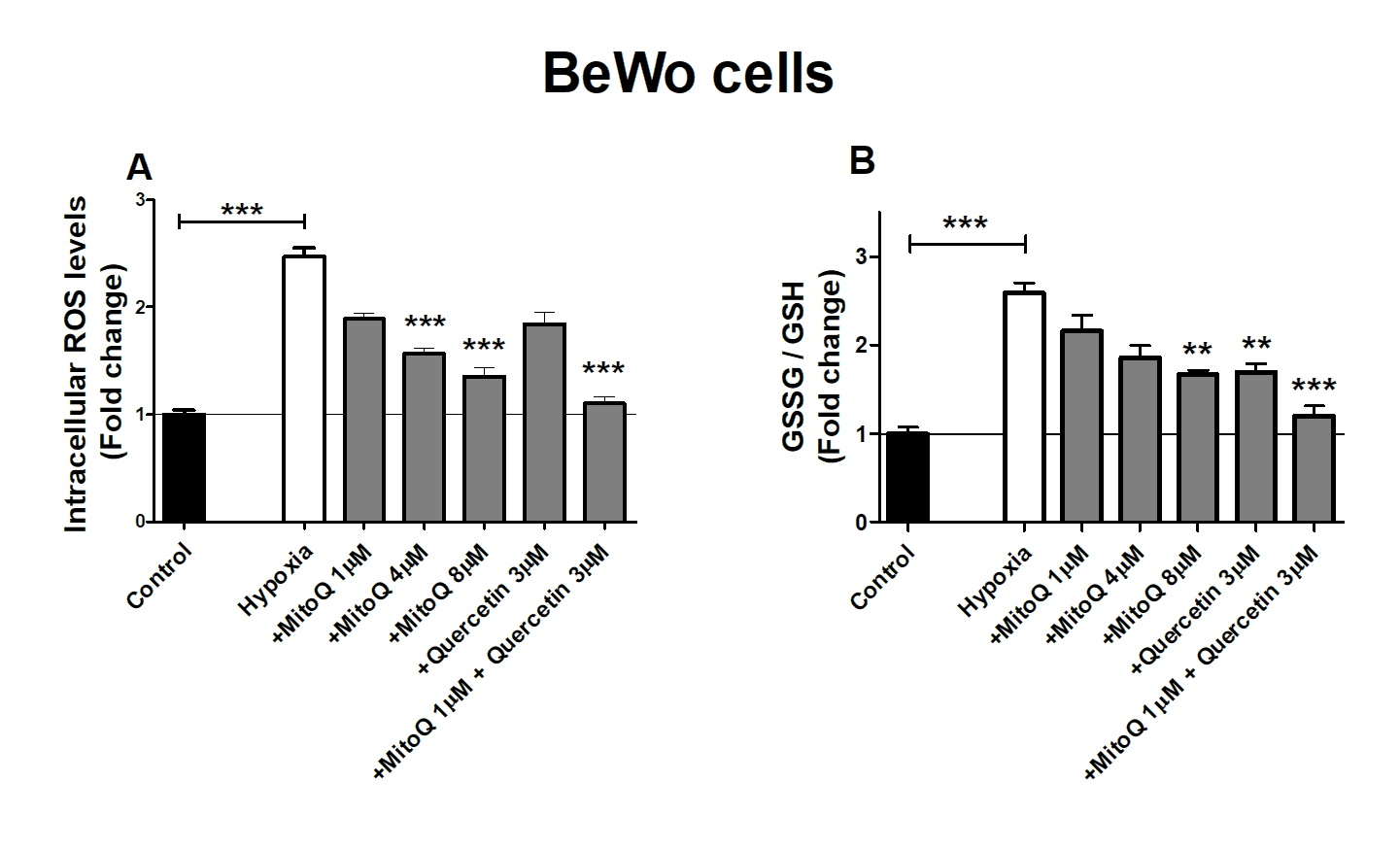

Supplement: S3 Fig — Intracellular ROS levels using DCFH-DA (A) and GSSG/GSH ratio (B) were assessed in trophoblasts exposed to normoxia (control), hypoxia, hypoxia + MitoQ (1, 4 or 8 μM), quercetin (3 μM) or MitoQ (1 μM) + quercetin (3 μM) for 3 h (DCFH-DA-assay: n = 6-12/experimental condition (n = 3 experiments) and GSSG/GSH-assay: n = 6, 2 and 2/experimental condition (n = 3 experiments)). Data are presented as fold change compared to the control and as mean with SEM. *p ≤ 0.05, **p ≤ 0.01 and ***p ≤ 0.001. DCFH-DA: 2’,7’-Dichlorodihydrofluorescein diacetate and GSSG: Glutathione disulfide, GSH: Glutathione. While placentae exposed to hypoxia did not show alterations in proinflammatory and apoptotic-related mRNA transcripts, mRNA levels of tumor necrosis factor-α (TNF-α), interleukin 6 (IL-6) and interleukin 8 (IL-8) as well as the pro-apoptotic ratio of Bcl-2associated X protein / anti-apoptotic B-cell lymphoma 2 (BAX/BCL-2) and protein levels of Cleaved Caspase-3 (17 + 15 KDa), were significantly increased in trophoblasts exposed for 24 h to hypoxia compared to the control condition (S4C–S4E Fig). (TIF) [file pone.0245155.s003.tif]

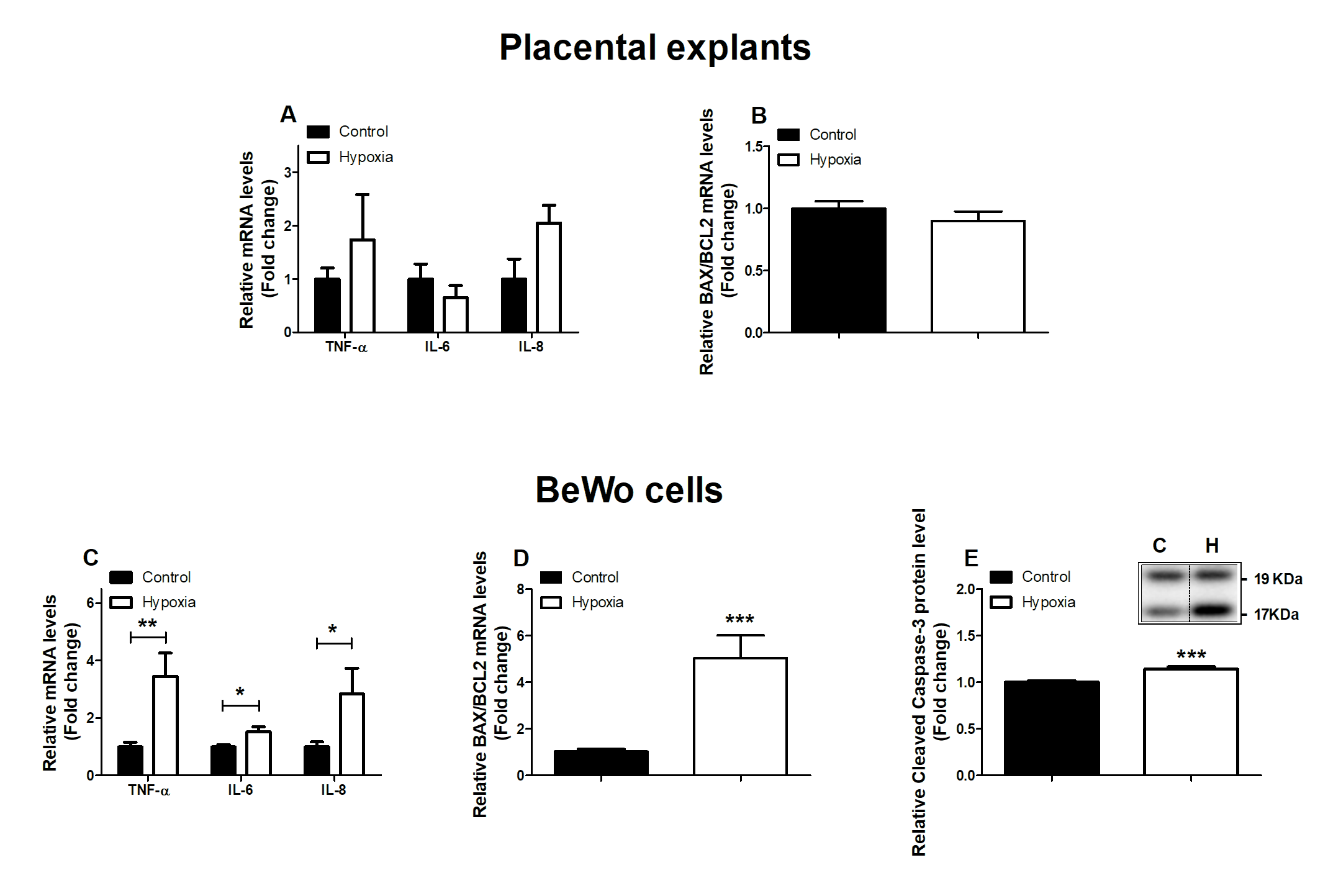

Supplement: S4 Fig — mRNA transcript levels of TNF-α, IL-6 and IL-8 (A) and ratio of BAX/BCL2 transcript levels (B) assessed in placental villous explants exposed to hypoxia or normoxia (control) for 3 h (n = 3) and mRNA transcript levels of TNF-α, IL-6 and IL-8 (C) and ratio of BAX/BCL2 transcript levels (D) and protein levels of Cleaved Caspase-3 (17 + 15 KDa) (E) assessed in trophoblasts exposed to normoxia (control) or hypoxia for 24 h (n = 3-6/experimental condition (n = 3 experiments)). Data are presented as fold change compared to the control and as mean with SEM. *p ≤ 0.05, **p ≤ 0.01 and ***p ≤ 0.001. TNF-α: Tumor necrosis factor α, IL-6: Interleukin 6, IL-8: Interleukin 8, BAX: pro-apoptotic Bcl-2-associated X protein and BCL: anti-apoptotic B-cell lymphoma 2. In contrast to the increased reliance on glycolysis in placental cells upon hypoxia, no differences were observed in activity and mRNA expression levels of the rate-limiting enzyme HADH of the FAO pathway in both placental villous explants and trophoblasts exposed to hypoxia compared to the control group (S5A–S5D Fig). (TIF) [file pone.0245155.s004.tif]

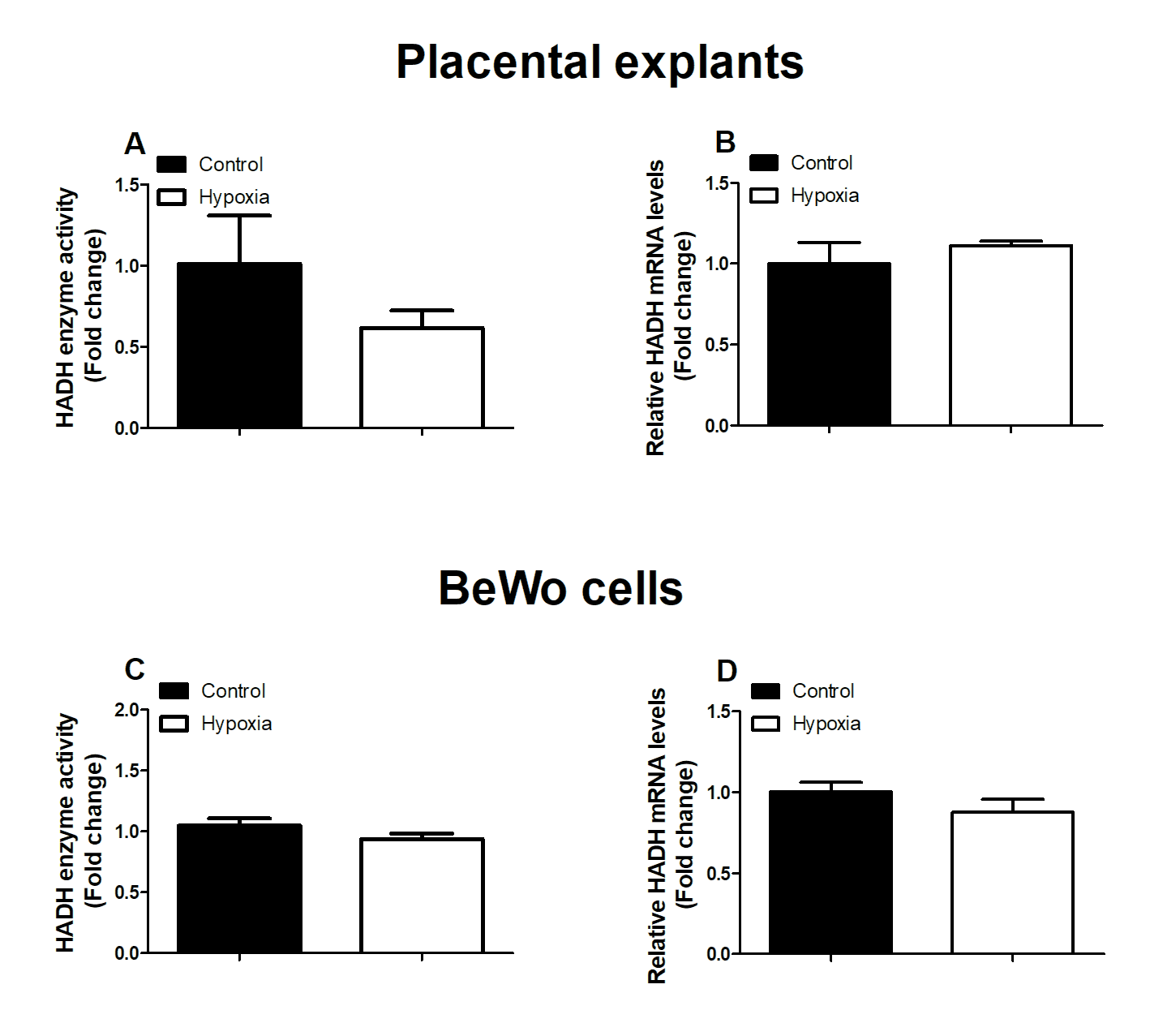

Supplement: S5 Fig — HADH enzyme activity (A) and mRNA transcript levels of HADH (B) assessed in placental villous explants exposed to hypoxia or normoxia (control) for 3 h (n = 3) and HADH enzyme activity (C) and mRNA transcript levels of HADH (D) assessed in trophoblasts exposed to normoxia (control) or hypoxia for 24 h (n = 3, 6 and 6/experimental condition (n = 3 experiments)). Data are presented as fold change compared to the control and as mean with SEM. HADH: 3-hydroxyacyl-CoA dehydrogenase. Protein levels of nuclear-encoded sub-units of the ETC including Ndufb8, Sdhb, UQCR2 and ATP5A of respectively complex I, II, III and V, were not affected in placental villous explants exposed for 3 h to hypoxia (S6A Fig). Also, related mRNA transcript levels of Ndufb3, Cycl-1, COXIV and COXII of respectively complex I, III, IV and IV, were not affected in placental villous explants after exposure to hypoxia (S6B Fig). Protein levels of UQCRC2 (complex III) as well as mRNA transcript levels of all investigated sub-units including mitochondrial encoded COXII were significantly decreased in trophoblasts upon 24h exposure to hypoxia (S6D–S6F Fig). (TIF) [file pone.0245155.s005.tif]

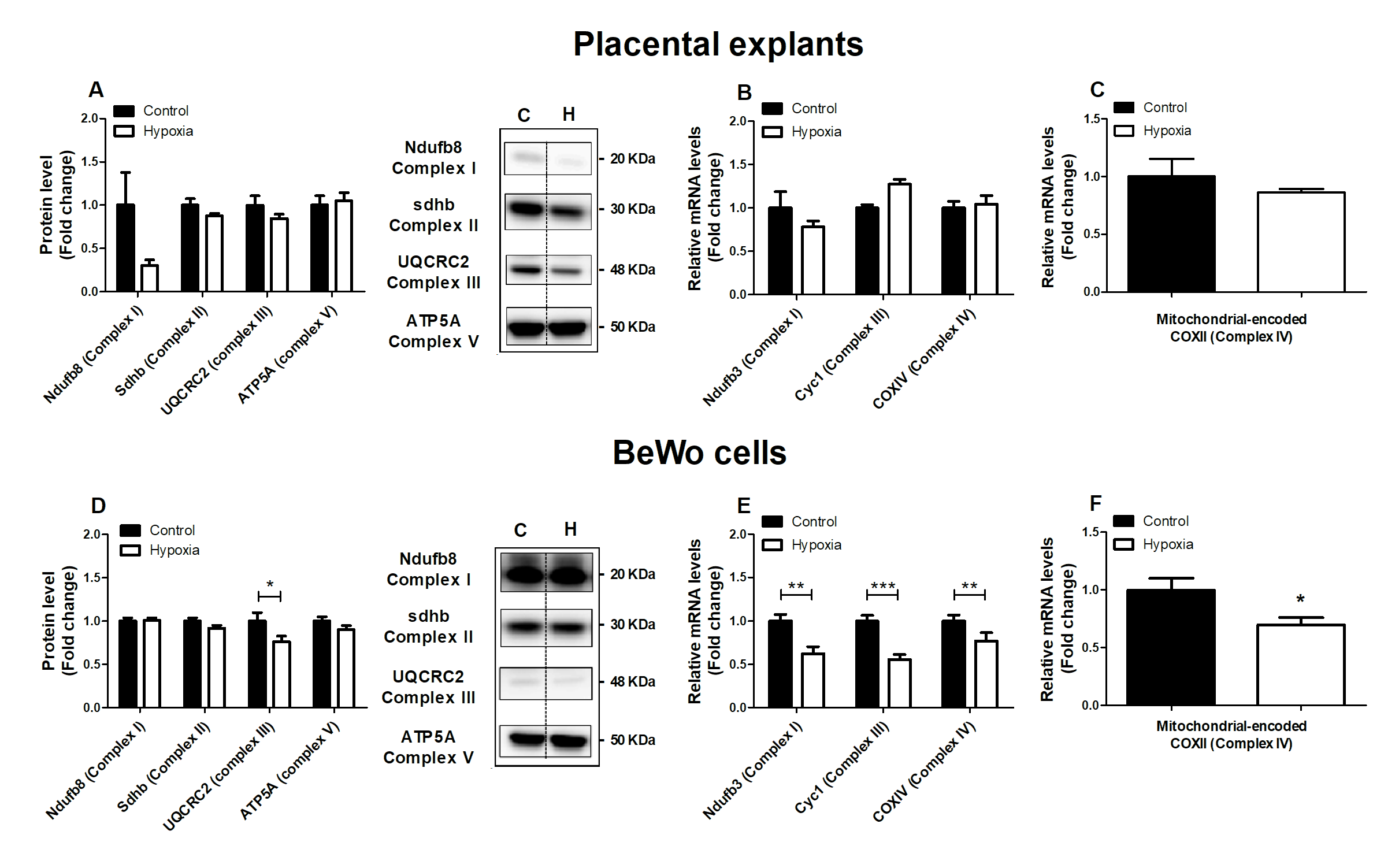

Supplement: S6 Fig — Protein levels of nuclear-encoded OXPHOS sub-units (Ndufb8, Sdhb, UQCRC2 and ATP5A respectively Complex I, II, III and V) (A) and mRNA transcript levels of nuclear-encoded OXPHOS sub-units (Ndufb, Cycl-1, COXIV and COXII respectively Complex I, III, IV and IV) (B) assessed in placental villous explants exposed to normoxia (control) or hypoxia for 3 h (n = 3) and protein levels of nuclear-encoded OXPHOS sub-units (Ndufb8, Sdhb, UQCRC2 and ATP5A respectively Complex I, II, III and V) (C) and mRNA transcript levels of nuclear-encoded OXPHOS sub-units (Ndufb, Cycl-1, COXIV and COXII respectively Complex I, III, IV and IV) (D) assessed in trophoblasts exposed to normoxia (control) or hypoxia for 24 h (n = 3-6/experimental condition (n = 3 experiments)). Representative immunoblots are shown and Western blots were corrected for total protein loading assessed by Ponceau S Staining with adjusted contrast equally applied to the whole photograph. Black boxes around the representative pictures indicate that they were cut from the same Western blot. Data are presented as fold change compared to the control and as mean with SEM. Ndufb3: NADH dehydrogenase [ubiquinone] 1 beta subcomplex subunit 3, Sdhb: Succinate dehydrogenase [ubiquinone] iron-sulfur subunit, UQCRC2: Cytochrome b-c1 complex subunit 2, ATP5A: ATP synthase F1 subunit alpha, Cyc1: Cytochrome C1, COXIV: Cytochrome c oxidase subunit IV and COXII: Cytochrome c oxidase subunit II. All investigated constituents of the autophagy in our study were unaltered in placental villous explants exposed for 3h to hypoxia compared to the control condition (S7A and S7B Fig). In cultured trophoblasts, 24 h hypoxia resulted in a significant decrease in autophagy-associated LC3BI and LC3BII protein levels while the ratio of LC3BI/LC3BII was significantly increased compared to the normoxic condition. In addition, while both protein as well as mRNA levels of GABA Type A Receptor Associated Protein Like 1 (GABARAPL1) were increased in resp [file pone.0245155.s006.tif]

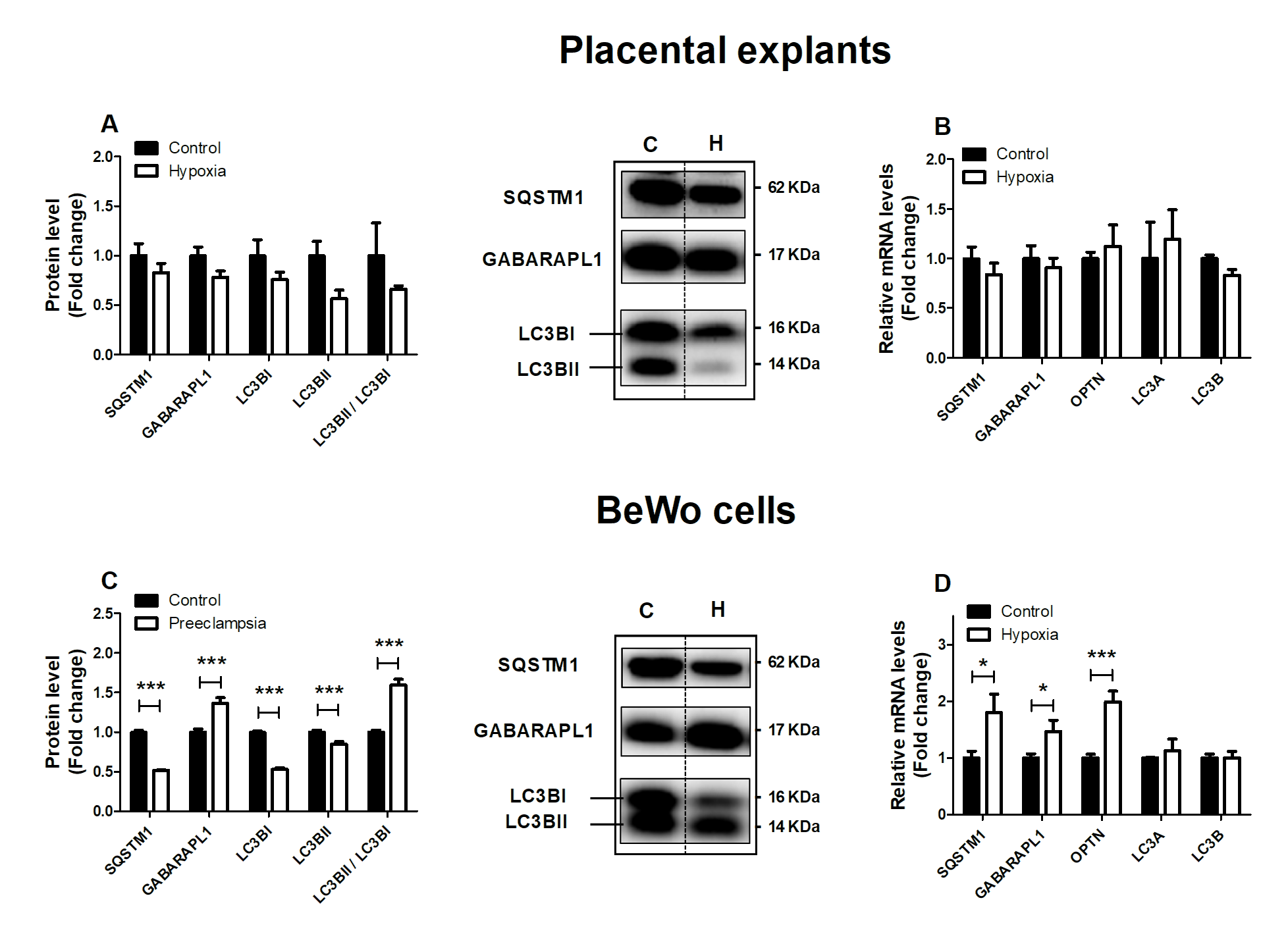

Supplement: S7 Fig — Autophagy-associated protein levels of SQSTM1, GABARAPL1, LC3BI, LC3BII and LC3BII / LC3BI ratio (A) and mRNA transcript levels of SQSTM1, GABARAPL1, OPTN and LC3B (B) assessed in placental villous explants exposed to normoxia (control) or hypoxia for 3 h (n = 3) and autophagy-associated protein levels of SQSTM1, GABARAPL1, LC3BI and LC3BII (C) and mRNA transcript levels of SQSTM1, GABARAPL1, OPTN and MAP1LC3A/B (D) assessed in trophoblasts exposed to normoxia (control) or hypoxia for 24 h (n = 3-6/experimental condition (n = 3 experiments)). Representative immunoblots are shown and Western blots were corrected for total protein loading assessed by Ponceau S Staining with adjusted contrast equally applied to the whole photograph. Black boxes around the representative pictures indicate that they were cut from the same Western blot. Data are presented as fold change compared to the control and as mean with SEM. *p ≤ 0.05 and ***p ≤ 0.001. SQSTM1: Sequestosome 1, GABARAPL1: GABA Type A Receptor Associated Protein Like 1, LC3B: Microtubule-associated protein 1 light chain 3 beta, OPTN: Optineurin and LC3A: Microtubule-associated protein 1 light chain 3 alpha. (TIF) [file pone.0245155.s007.tif]
