# Supplementary material for: Hypoxia-induced mitochondrial abnormalities in cells of the placenta
Source: PLoS One. 2021 Jan 12;16(1):e0245155. doi: 10.1371/journal.pone.0245155 (PMC7802931; doi:10.1371/journal.pone.0245155)
Supplement: S1 Table — (DOCX) [file pone.0245155.s008.docx]

**S1 Table. Primers used for qPCR**

| **Target** | **Sense primer (5’-3’)** | **Antisense primer (3’-5’)** |
| --- | --- | --- |
| *Cat1* | GATGTGCATGCAGGACAATCAG | GCTTCTCAGCATTGTACTTGTCC |
| *XO* | AGAAAGTTGGGGCTGAGTGG | GCAGGCATTGGCAGAAAAGT |
| *SOD1* | GGTCCTCACTTTAATCCTCTAT | CATCTTTGTCAGCAGTCACATT |
| *MnSOD2* | TGGACAAACCTCAGCCCTAACG | TGATGGCTTCCAGCAACTCCC |
| *TNF-α* | ATGCCTTTAGATGTGAGCTAACAGTAGGTA | CGTACAGCCATCAAAAAGGGACAC |
| *BAX* | GGTCTTTTTCCGAGTGGCAG | CACAGGGCCTTGAGCAC |
| *BCL-2* | GTCTTTTTCCGAGTGGCAGC | GTAGAAAAGGGCGACAACCC |
| *RPL13A* | CCTGGAGGAGAAGAGGAAAGAGA | TTGAGGACCTCTGTGTATTTGTCAA |
| *COXII* | ACCTGCGACTCCTTGACGTT | GGGGGCTTCAATCGGGAGTA |
| *CS* | GATGTGTCAGATGAGAAGTTACGAGACT | TGGCCATAGCCTGGAACAA |
| *HADH* | TGGCTTCCCGCCTTGTC | TGGAGCCGGTCCACTATCTTC |
| *HKII* | GTAAATACAGTGGATCTCAATCTTCGGG | CAAGGATTTGAGATGATTCGCTATTCA |
| *GLUT1* | TCTGGGCTGCCGGGTTCTAG | TTTGCAGGCTCCCACAGGC |
| *Cycl1* | GCATTCGGAGGGGTTTCCAG | CCGCATGAACATCTCCCCA |
| *COXIV* | CCATGGATGAGAAAGTCGAGT | CGTTCGAGCCCCTGTTCA |
| *PGC-1α* | AAGCCACTACAGACACCGC | TCGTAGCTGTCATACCTGGG |
| *PGC-1β* | CAGAACAAGGAGGCGGAGGTC | AGGTCCAAGTTTGCGAAGC |
| *NRF1* | GCACCTTTGGAGAATGTGGT | CTGGGATAAATGCCCGAAG |
| *NRF2* | CTCACCTGGGAACAGAACAGGAA | ACCCAAGAAATGCAGTCTCGAGC |
| *ERRα* | TGCTGCTCACGCTACCGCTC | TCGAGCATCTCCAAGAACAGC |
| *Tfam* | GAAAGATTCCAAGAAGCTAAGGGTGATT | TCCAGTTTTCCTTTACAGTCTTCAGCTTTT |
| *PPARα* | CAGAACAAGGAGGCGGAGGTC | AGGTCCAAGTTTGCGAAGC |
| *PPARδ* | TGACCAAAAAGAAGGCCCGC | GTCGTGGATCACAAAGGGCG |
| *SQSTM1* | GGTGCACCCCAATGTGATCT | CGCAGACGCTACACAAGTCG |
| *PINK1* | GAAAGCCGCAGCTACCAAGA | AGCACATTTGCGGCTACTCG |
| *PARK2* | GGTTTGCCTTCTGCCGGGAATG | CTTTCATCGACTCTGTAGGCCTG |
| *FUNDC1* | GAAACGAGCGAACAAAGCAG | GCAAAAAGCCTCCCACAAAT |
| *BNIP3* | AGCGCCCGGGATGCA | CCCGTTCCCATTATTGCTGAA |
| *BNIP3L* | CTGCGAGGAAAATGAGCAGTCTCT | GCCCCCCATTTTTCCCATTG |
| *OPTN* | AAGGAGCAACTGGCATTGCA | TCTCCATCAAGGACTGCCTG |
| *GABARAPL1* | ATCGGAAAAAGGAAGGAGAAAAGATC | CAGGCACCCTGGCTTTTGG |
| *LC3A* | CCTGGACAAGACCAAGTTTTTG | GTCTTTCTCCTGCTCGTAGATG |
| *LC3B* | ACCATGCCGTCGGAGAAGAC | TCTCGAATAAGTCGGACATCTTCTACTCT |
| *Fis-1* | CCTGGTGCGGAGCAAGTACAA | TCCTTGCTCCCTTTGGGCAG |
| *DNM1L* | CGACTCATTAAATCATATTTTCTCATTGTCAG | TGCATTACTGCCTTTGGCACACT |
| *Mfn1* | CTGAGGATGATTGTTAGCTCCACG | CAGGCGAGCAAAAGTGGTAGC |
| *Mfn2* | TGGACCACCAAGGCCAAGGA | TCTCGCTGGCATGCTCCAC |
| *OPA1* | TACCAAAGGCATTTTGTAGATTCTGAGTT | GCATGCGCTGTATACGCCAA |
| *IL-6* | CCTGAACCTTCCAAAGATGGC | CACCAGGCAAGTCTCCTCATT |
| *IL-8* | TTAGAACTATTAAAACAGCCAAAACTCCACA | CAAGTTTCAACCAGCAAGAAATTACTAATATTG |
| *Ndufb3* | ACAGACAGTGGAAAATTGAAGGG | GCCCATGTATCTCCAAGCCT |

Cat1: Catalase-1, XO: Xanthine oxidase, SOD1: Superoxide dismutase 1, MnSOD2: Manganese-dependent superoxide dismutase, TNF-α: Tumornecrosefactor α, BAX: Pro-apoptotic Bcl-2-associated X protein, BCL: Anti-apoptotic B-cell lymphoma 2, RPL13A: Ribosomal Protein L13a, COXII: Cyclo-oxygenase 2, CS: Citrate synthase, HADH: 3-hydroxyacyl-CoA dehydrogenase, HKII: Hexokinase, GLUT1: Glucose transporter 1, Cyc1: Cytochrome C1, COXIV: Cytochrome c oxidase subunit IV, PGC-1α: Peroxisome proliferator-activated receptor gamma coactivator 1-alpha, PGC-1β: Peroxisome proliferator-activated receptor gamma coactivator 1-beta, NRF1: Nuclear respiratory factor 1, ERRα: Estrogen-related receptor alpha, Tfam: Transcription factor A, PPARα: Peroxisome proliferator-activated receptor alpha, PPARδ: Peroxisome proliferator-activated receptor delta, SQSTM1: Sequestosome 1, PINK1: PTEN-induced kinase 1, PARK2: Parkin, FUNDC1: FUN14 domain containing 1, BNIP3: BCL2/adenovirus E1B 19 kDa protein-interacting protein 3, BNIP3L: BCL2/adenovirus E1B 19 kDa protein-interacting protein 3-like, OPTN: Optineurin, GABARAPL1: GABA Type A Receptor Associated Protein Like 1, LC3A: Microtubule-associated protein 1 light chain 3 alpha, LC3B: Microtubule-associated protein 1 light chain 3 beta, Fis-1: Fission 1 protein, DNM1L: Dynamin-related protein 1, Mfn1: Mitofusin-1, Mfn2: Mitofusin-2, OPA1: Optic atrophy protein 1. IL-6: Interleukin 6, IL-8: Interleukin 8 and Ndufb3: NADH oxidoreductase subunit B3.
