# Supplementary material for: Hypoxia-induced mitochondrial abnormalities in cells of the placenta
Source: PLoS One. 2021 Jan 12;16(1):e0245155. doi: 10.1371/journal.pone.0245155 (PMC7802931; doi:10.1371/journal.pone.0245155)
Supplement: Attachment — Submitted filename: Response to Reviewers.docx [file pone.0245155.s009.docx]

*Partha Mukhopadhyay, Ph.D.*

*Academic Editor*

*PLOS ONE*

*8-11-2020*

Dear dr. Partha Mukhopadhyay,

Manuscript reference: Ms. No. PONE-D-20-05832

Please find attached a revised version of our manuscript “***Hypoxia-induced mitochondrial abnormalities in cells of the placenta***”, which we would like to resubmit for publication as a research paper in *PLOS ONE*.

The comments of the reviewers are implemented in the revised manuscript. In the following pages are our point-by-point responses to each of the comments of the reviewers. The blot/gel image data are now included as supporting information (S1_Fig).

Changes in the revised manuscript are shown using red highlight for changes in the document “Manuscript with track changes”. We hope that the revisions in the manuscript will be sufficient to make our manuscript suitable for publication in *PLOS ONE*.

We look forward hearing from you at your earliest convenience.

Yours sincerely,

Philippe Vangrieken, PhD

E-mail: p.vangrieken@maastrichtuniversity.nl

**General comment 1. Please ensure that your manuscript meets PLOS ONE's style requirements, including those for file naming. The PLOS ONE style templates can be found at http://www.plosone.org/attachments/PLOSOne_formatting_sample_main_body.pdf and http://www.plosone.org/attachments/PLOSOne_formatting_sample_title_authors_affiliations.pdf**

Author response: We have checked the requirements and the manuscript now meets all guidelines for PLOS One.

**General comment 2. Thank you for specifying in your ethics statement that participant consent was verbal. Please also specify: 1) whether the ethics committee approved the verbal consent procedure, 2) why written consent could not be obtained, and 3) how verbal consent was recorded.**

Author response: The following statement is added to the manuscript (LINE 72-79): “*The local medical ethics committee Maastricht University Medical Center+ approved the procedures and all procedures on placentae were performed in accordance with the guidelines for experiments on human material (Maastricht University Medical Center+, Maastricht, the Netherlands; No. 16-4-047) by which placental samples were either collected as part of a prospective study for which participants gave written and verbal consent or collected as “biological waste” from discarded tissue if participants did not verbally object to this happening, as documented by the health care provider in their medical files.”*

**General comment 3: Thank you for stating the following in the Acknowledgments Section of your manuscript: "*This study was supported by NUTRIM Graduate Program. There was no additional external funding received for this study*." We note that you have provided funding information that is not currently declared in your Funding Statement. However, funding information should not appear in the Acknowledgments section or other areas of your manuscript. We will only publish funding information present in the Funding Statement section of the online submission form.**

Author response: The funding statement is removed from the Acknowledgments section.

**General comment 4: Please remove any funding-related text from the manuscript and let us know how you would like to update your Funding Statement. Currently, your Funding Statement reads as follows: "*The funders had no role in study design, data collection and analysis, decision to publish, or preparation of the manuscript*."**

Author response: We have removed funding-related text from the manuscript. We agree with the current funding statement and feel it is not necessary to update.

**General comment 5: PLOS ONE now requires that authors provide the original uncropped and unadjusted images underlying all blot or gel results reported in a submission’s figures or Supporting Information files. This policy and the journal’s other requirements for blot/gel reporting and figure preparation are described in detail at https://journals.plos.org/plosone/s/figures#loc-blot-and-gel-reporting-requirements and https://journals.plos.org/plosone/s/figures#loc-preparing-figures-from-image-files. When you submit your revised manuscript, please ensure that your figures adhere fully to these guidelines and provide the original underlying images for all blot or gel data reported in your submission. See the following link for instructions on providing the original image data: https://journals.plos.org/plosone/s/figures#loc-original-images-for-blots-and-gels.**

Author response: The file containing original uncropped and unadjusted images underlying all blot or gel results is now added as supporting file (S1 Fig).

**General comment 6: In your cover letter, please note whether your blot/gel image data are in Supporting Information or posted at a public data repository, provide the repository URL if relevant, and provide specific details as to which raw blot/gel images, if any, are not available. Email us at plosone@plos.org if you have any questions.**

Author response: It is now stated in the cover letter.

**General comment 7: We note that you have included the phrase “*data not shown*” in your manuscript. Unfortunately, this does not meet our data sharing requirements. PLOS does not permit references to inaccessible data. We require that authors provide all relevant data within the paper, Supporting Information files, or in an acceptable, public repository. Please add a citation to support this phrase or upload the data that corresponds with these findings to a stable repository (such as Figshare or Dryad) and provide and URLs, DOIs, or accession numbers that may be used to access these data. Or, if the data are not a core part of the research being presented in your study, we ask that you remove the phrase that refers to these data.**

Author Response: We now have added a new figure (S2 Fig) to the revised manuscript.

Review Comments to the Author

**Reviewer #1: In the current study "Hypoxia-induced mitochondrial abnormalities in cells of the placenta" the authors analyzed the mitochondrial features in placenta explants and a trophoblast cell line under hypoxia. Here are some major concerns:**

**Reviewer comment 1: The authors provided evidence that the hypoxic trophoblast cells have higher molecular capacity of glycolysis, which is a common phenotype in most cells under hypoxia. Have the authors analyzed the metabolism pattern of the cells with oxygen consumption, pH change and glycolysis product quantification? How is glycolysis linked to the onset of PE?**

Author response: We thank the reviewer for this valid comment and suggestions. In response to the comment related to measuring O_2_ consumption, we would like to indicate that respiration measurements, due to inherent technical reasons, cannot be performed in a hypoxic environment. Also, exposing the cells to ambient oxygen levels after a hypoxic incubation (as would happen upon measuring O_2_ consumption in our cells after hypoxic incubation) would induce acute changes in metabolism which we want to avoid. Therefore, although with respirometry ECAR (extra-cellular acidification rate; as a measure for glycolysis) is possible to detect, we feel that respiration measurements may not be ideal in our experimental hypoxia setting. However, as suggested by the reviewer we have now measured lactate levels, as an established end-product of glycolysis, in lysates of BeWo cells after 24 h exposure to hypoxia and found significantly higher levels (+ 50%) in hypoxia-exposed cells compared to cells cultured in normoxic conditions. This confirms our previous observations related to hypoxia-induced upregulation of the glycolytic pathway. We have now included this new data in the revised manuscript (Fig 3G). In addition, as the HEPES buffer and the F12 media that we used for BeWo cell culture are buffered, we were not able to detect reliable changes in the pH of cell supernatant after exposure of the cells to hypoxia. With regard to the question of the reviewer ‘’ *How is glycolysis linked to the onset of PE*’’ we have included the following text in the discussion of the revised manuscript (Line 609-615): “*The increased reliance on glycolysis found in cells of PE placentae or upon hypoxia may be a consequence of the decreased mitochondrial content and impaired functioning of the electron transport chain and thus capacity for producing ATP. This shift to anaerobic respiration may also be a protective response of the placenta to the impaired placental perfusion in PE and so sparing O_2_ for the growing fetus. It is thus expected that increased glycolysis is more likely a consequence than involved in the onset of PE* (Postigo L, et al. 2009)*.*”

**Reviewer comment 2: The authors want to link the dysfunction of mitochondria with cell apoptosis and mitophagy. However, gene expression analysis with mRNA and western blot protein quantification is not enough to make a conclusion. The authors need to analyze apoptosis with TUNEL staining and caspase-3 cleavage. Also, the authors need to confirm mitophagy with electromicroscopy.**

Author response: We agree with the reviewer that quantification of transcript and protein levels of constituents of the mitophagy machinery that we measured in our manuscript, is not sufficient to make hard conclusions regarding active breakdown of mitochondria through the process of mitophagy. However, the changes in the expression/abundance of these proteins that we observe have been associated with activation of mitophagy as evidenced by several papers that we reference in the revised manuscript (LINE 642). Unfortunately, we are currently not able to perform EM in our institution due to reduced capacity in light of COVID-19 measures. To meet the reviewers’ concerns, in the revised manuscript we have now nuanced our conclusions regarding the activity of mitophagy in response to hypoxia (LINE 649-652 and 659-665). We now emphasize that our observations are merely suggestive of changes in the molecular control of mitophagy and refrain from hard conclusions regarding the activity of mitophagy. We hope this is sufficient to meet the reviewers’ concerns.

With regard to the suggestions regarding apoptosis, we agree with the reviewer that solely showing changes in the relative transcript abundance of bax/bcl-2 (although robustly induced by approximately 6-fold by hypoxia) is insufficient evidence of apoptosis. Although we would like to highlight that apoptosis was not a primary read-out in our experiments (and therefore is only presented as part of S4 Fig). However, to address the reviewers’ comment, also in light of another comment of the reviewer, we have now assessed mitochondrial membrane potential (a known trigger of cellular apoptosis; Barroso, 2006, (LINE 555-557, Fig 2F) and found a significant loss of mitochondrial membrane potential in response to hypoxia, which is in line with a potential induction of apoptosis (Fig 2F). Also, we have now included more evidence from literature evidencing that trophoblasts undergo apoptosis under hypoxic conditions (LINE 549-557). In addition, we have assessed protein levels of cleaved caspase-3 by Western blot and found significantly increased levels in response to hypoxia. Collectively, we feel that these observations strengthen the notion of hypoxia-induced apoptosis in our cell cultures. We have added this data in S4 Fig E of the revised manuscript and made reference to relevant literature in the discussion of the revised manuscript (LINE 549-557)).

**Reviewer comment 3: The authors tried to analyze the biogenesis, fission and fusion of mitochondria and provide explanation for the dropped number of mitochondria. All these gene expression results need to be paired with morphological study with electromicroscopy to make a conclusion. Also, the morphological mitochondrial changes and damages can be seen in such experiments.**

Author response: The reviewer is correct in his/her observation that solely measuring protein and mRNA levels of key regulators of mitochondrial biogenesis and mitochondrial fusion/fission are insufficient to draw hard conclusions regarding the activity of these dynamic processes. However, with regard to mitochondrial biogenesis, the fact that we observe reduced mRNA levels of nuclear- as well as mitochondrial-encoded OXPHOS subunits in trophoblasts exposed to hypoxia (S6 Fig E, F) does suggest an impairment in the activity of mitochondrial biogenesis (as also stated in the discussion of the manuscript (LINE 622-629) as the expression of these genes is tightly controlled by the same key regulators of mitochondrial biogenesis that we measured. More specifically, Tfam and ERRα (both significantly reduced on the mRNA and protein level upon hypoxia in our study; Fig 4) have been convincingly demonstrated to control expression of mitochondrial-encoded and nuclear OXPHOS genes respectively. We feel that this strengthens our suggestion of hypoxia-induced impairment of mitochondrial biogenesis and has added this notion to the revised version of the discussion (LINE 625-629). In addition, we feel that the dynamic nature of the processes of fusion/fission and mitochondrial biogenesis also limits accurate quantitative detection of these processes using electron microscopy. Although EM could shed some more light on this, we are currently unable to perform such analyses due to reduced capacity in light of COVID-19 measures. Therefore, to address the reviewers’ comment, we have nuanced our conclusions regarding the impact of hypoxia on these processes in the revised version of the discussion (pg 25). We have also suggested this notion as a future perspective of the study in the revised version of the manuscript.

**Reviewer comment 4: The authors should analyze the mitochondrial membrane potential.**

Author response: As suggested by the reviewer, we have now measured mitochondrial membrane potential in BeWo cells upon exposure to hypoxia for 24 h (Fig 2F). In line with our expectations, mitochondrial membrane potential was significantly reduced upon exposure to hypoxia. We have also added some text to the discussion of the revised manuscript addressing this (LINE 555-557).

**Reviewer #2: The authors have done an incredible job of pursuing and presenting their work effectively. The rationale, experimental design, and results are convincing. However, the manuscript presents specific gaps addressing which will improve this work substantially.

Major comments:

Reviewer comment 1: I like that the authors have provided extensive details of the experimental techniques and assay procedures that they used in the project. However, I would like to point out that the table consisting of the list of primary antibodies should be part of the main manuscript, not a supplemental table. The table should provide details of the exact dilutions used for the specific primary antibodies used in the western blots. Currently, the table lacks the dilutions for each antibody.**

Author response: We would like to thank the reviewer for his/her compliments. With regard to the first comment, the table with the primary antibodies and related dilutions is now included in the main body of the revised version of the manuscript (pg 11, Table 1).

**Reviewer comment 2: Throughout the manuscript, the authors have used a 3-hr window for subjecting the placental explants to hypoxic stress. They do not provide any explanation on the rationale until we reach the discussion (lines 465-467) about the selected number of hours. First, the authors should give the justifications of their experimental conditions at the beginning – in the relevant results section before stating the precise outcomes of the experiments.**

Author response: We agree with the reviewer that this should be discussed/mentioned earlier in the manuscript. Therefore, as suggested by the reviewer, the explanation of the rationale of the exposure times of placental explants and BeWo cells to hypoxic conditions is now moved to the start of the results section (LINE 292-298).

**Reviewer comment 3: Second, they should consider providing experimental evidence of placental tissue deterioration rather than only providing references. They should consider presenting GSSG/GSH, and TEAC fold changes when the placental explants are subjected to hypoxic stress over 2, 4, 5, and/or 7 hours, along with 3 hours, to reveal the changes observed with these parameters, concomitant with reported tissue deterioration. This data will provide experimental rigor to their findings and will substantiate their scientific decision to choose 3 hours for placental explants.**

Author response: We understand the point the reviewer is raising. However, unfortunately, at the time we did not include the suggested measurements in our initial experiments with placental explants that we freshly isolated from deliveries. However, importantly, we did assess LDH release in the explant supernatant to provide direct experimental evidence (in our own experiments) of viability of the explants which we feel was the most important quality control to include. This confirmed no tissue deterioration and cellular apoptosis in our placental villous explants, which could otherwise, besides hypoxia, be a driver affecting GSSG/GSH and TEAC. We hope that this addresses the concerns of the reviewer. We have added some text to the results of the revised manuscript to describe this more clearly (pg 14, LINE 301-309).

**Reviewer comment 4: In Fig 2, the authors present the effect of hypoxic stress on mtDNA copy number and citrate synthase (CS) enzyme activity on the placental explants and BeWo cells. However, they have presented the effect of mitochondrial antioxidants MitoQ and Quercetin only on BeWo cells. Have the authors performed the rescue experiment on the placental explants too? These results will consolidate their findings with the cultured trophoblasts. The authors should consider presenting the data as part of Fig 2.**

Author response: We agree that it would have been nice to perform such a rescue experiment using MitoQ and Quercetin in hypoxia-exposed placental explants, but we have not performed such an experiment as, after the experiments in the placental explants, we were subsequently interested in pursuing to investigate whether the observed changes were trophoblast-specific. Furthermore, during our experimental procedure, the placental explant is not perfused and, as a consequence, the uptake and homogenous distribution of MitoQ and quercetin are difficult to predict, which may result in skewing of results. We have added the suggestion of the reviewer in the discussion (LINE 679-682).

**Reviewer comment 5: There are significant discrepancies in results between the placental explants and the BeWo cell line. The authors have not explained each sub-figure of every figure in detail.**

Author response: We agree with the reviewer. The discrepancies found between placental explants and BeWo cells, which are indeed present in several read-outs are now further discussed through the discussion section (pg 21-25 and LINE 629-633).

**Reviewer comment 6: Also, the discussion part of the manuscript hardly refers back to specific figures and results while providing the rationales of their overall findings. The discussion has to be streamlined with references to particular figures with explanations to every data obtained in them.**

Author response: We agree with the reviewer and we now specifically refer back to specific figures and results found in our study along with relevant references.

**Minor comments

Reviewer comment 1: Figs 2 through 6 and supplemental Figs 4 and 5 present western blots that are lacking the molecular weight markers for each panel, they should be included in the respective figures.**

Author response: The molecular weight is now added to the figures.

**Reviewer comment 2: The authors mention that they have used the Ponceau Staining image for normalization of the band signal of each protein on the blots, so, they should consider including the Ponceau stained membrane image in the figures.**

Author response: The Ponceau-stained membrane image is now presented in S1 Fig.
